# A large 4 K dataset of supermicrosurgical anastomosis at the 0.2 mm scale

**DOI:** 10.1038/s41597-026-07874-0

**Published:** 2026-07-21

**Authors:** Jing Liang, Jingyuan Fan, Aihemaitijiang Yusufu, Jianxi Hou, Dewei Zhao, Qingtang Zhu

**Affiliations:** 1 Department of Microsurgery, Trauma and Hand Surgery, the First Affiliated Hospital of Sun Yat‐sen University, Guangzhou, 510080 Guangdong China; 2 Department of Trauma and Microreconstructive Surgery, the First Affiliated Hospital of Xinjiang Medical University, Urumqi, 830000 Xinjiang China; 3 Department of Microsurgery, Zhengzhou Renji Hospital, Zhengzhou, 450000 Henan China; 4 Department of Orthopedics, Affiliated Zhongshan Hospital of Dalian University, Dalian, 116000 Liaoning China; 5 Guangdong Provincial Key Laboratory for Orthopedics and Traumatology, Guangzhou, 510080 Guangdong China

## Abstract

Supermicrosurgery, involving vessels smaller than 0.8 mm, is an important emerging field in reconstructive surgery. Its development is limited by the lack of suitable training resources and the current performance of microsurgical robotic systems. Existing robots provide limited intelligent assistance, and their anastomosis procedures are significantly slower than manual techniques. Here, we present the Microsurgical Silicone Tube Anastomosis dataset (MSTA), a large-scale 4 K video dataset of 0.2-mm silicone tube anastomosis procedures. The dataset includes 452 procedures performed by 571 operators from 158 hospitals across 28 provinces in China, with a total of 40.77 hours of 4 K video (3,840 × 2,160 pixels). All procedures were performed using standardized microsurgical instruments and imaging settings to ensure consistency and comparability. The participants represented a wide range of experience levels, with completion times ranging from 125 to 600 seconds, providing a broad spectrum of technical performance for analysis. Each procedure is accompanied by quantitative metadata, including completion time and expert-assessed performance scores based on predefined criteria. These data enable objective assessment of technical proficiency and analysis of performance-related factors. In addition, the dataset contains 47,452 segmentation annotation surgical frames, supporting computer vision studies. The MSTA dataset addresses the current gap in supermicrosurgical training data, and supports both surgical-robotics development and human training via objective performance assessment. Its scale and standardized design provide a reliable basis for building intelligent assistance systems and establishing benchmarks for skill evaluation in supermicrosurgery.

## Background & Summary

**Fig. 1:**
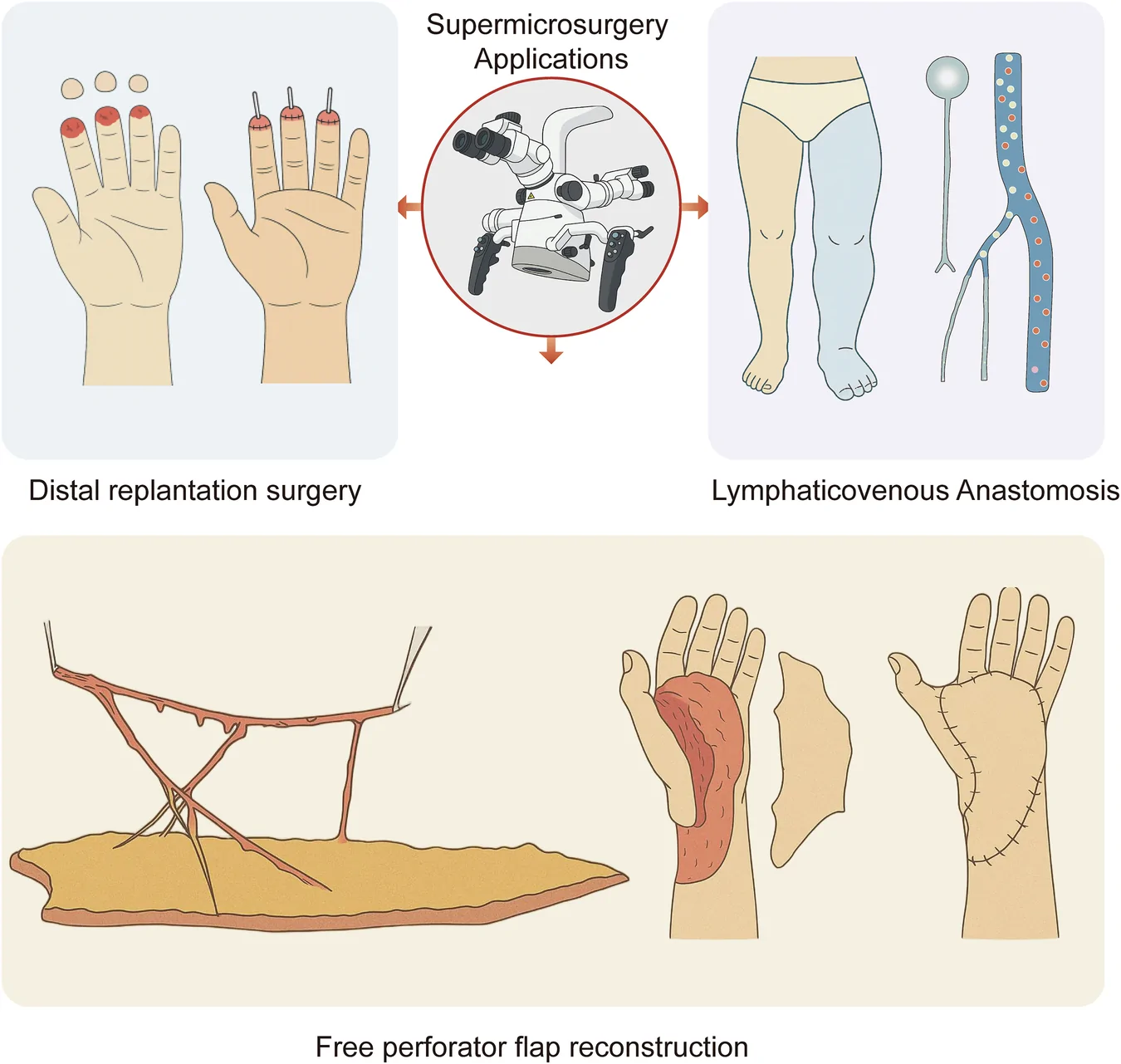
Clinical applications of supermicrosurgery. Supermicrosurgery, performed under high magnification (center), allows reliable manipulation of vessels < 0.8 mm. Top left: distal fingertip replantation with anastomosis of tiny digital arteries and veins. Top right: lymphaticovenous anastomosis for lymphedema, connecting lymphatic vessels to subdermal venules to restore drainage. Bottom: free perforator flap reconstruction, from harvesting a thin perforator-based flap to hand defect coverage and primary donor-site closure, illustrating refined submillimeter reconstruction with improved function and contour.

With advances in microsurgical techniques, anastomoses involving vessels smaller than 0.8 mm in diameter can now be achieved through supermicrosurgical procedures^1–3^. Since their introduction by Koshima *et al*. in the 1990s^4,5^, supermicrosurgery has made continuous progress in lymphaticovenous anastomosis for lymphedema^6^, perforator flap reconstruction^4,7^, and fingertip replantation^8^ (Fig. 1). The core of this technique lies in managing transparent^9^, fragile^10^, submillimeter vessels with 11-0 to 12-0 sutures and a 50-μm needle^11^, typically under up to 50 × magnification^12^, employing single-handed suturing guided by needle-tip tactile feedback^10^. Clinical success rates have reached nearly 98% in applications such as head and neck soft tissue defect reconstruction^13^. This approach has demonstrated advantages over traditional microsurgical methods in terms of functional restoration and reduction of complications, and has become one of the most significant advances in microsurgical reconstruction^14^.

Supermicrosurgery represents a significant advance in reconstructive and lymphatic surgery, but its clinical adoption remains limited by two main challenges. First, physiological hand tremor restricts surgical precision, and quantitative studies report mean tremor amplitudes of 100–200 μm during microsurgical tasks^15–18^, a scale comparable to the 0.2 mm diameter of lymphatic and microvascular channels. This scale overlap makes supermicrosurgery especially difficult, as it demands exceptionally high accuracy. Another barrier is the slow learning curve^19,20^, which hampers wider clinical adoption of supermicrosurgery techniques. Consistent success at this level requires prolonged, structured training. Individual differences in perceptual acuity and tactile proficiency play an important role in skill development and performance progression^21,22^.

Currently, solutions to these challenges follow two development directions. First is human-centered: video-based teaching, standardized curricula, and high-fidelity simulators are used to accelerate skill acquisition and improve training experiences^23,24^. Second is technology-driven: microsurgical robotic systems (Symani^25^ and MUSA-2^26^) paired with artificial intelligence (AI) algorithms are designed to counteract physiological tremor and maintain consistent execution. However, current microsurgical robotic platforms typically require a longer anastomosis time than manual operation, thereby extending overall procedure duration^27^. Robotic systems also lack autonomous decision-making, limiting their capacity to provide truly adaptive intelligent assistance.

Therefore, establishing a standardized, high-quality supermicrosurgical dataset that supports both surgical training and the development of machine learning algorithms represents an important step toward advancing capabilities in this field for human and AI. Existing microsurgical video datasets, such as MISAW^28^ and CUHK-MRG^29^, primarily focus on conventional microsurgical procedures and do not encompass the supermicrosurgical domain at the submillimeter scale.

To address this gap, we built a detailed dataset capturing complete anastomoses of 0.2 mm vessel models using standardized silicone tubes. The collection includes approximately 40.77 hours of high-definition video and 146,770 high-resolution still images (3840 × 2160, 4 K Ultra HD, 16:9). Data were contributed by 571 operators from 158 hospitals across 28 provinces. All recordings used the same microscopy platforms, instruments, and anastomotic materials to ensure procedural consistency. Each procedure was evaluated by experts using standardized performance scores, enabling objective assessment of technical skills. The Microsurgical Silicone Tube Anastomosis (MSTA) dataset is also designed to support human training and algorithm development. In education, the standardized, annotated recordings provide measurable benchmarks and allow objective competency assessment, helping to accelerate skill acquisition by young surgeons. In computational science and robotics research, the MSTA dataset offers precise, real-world training data for applications including automatic phase recognition, real-time instrument tracking, tremor characterization, and autonomous robotic control.

## Materials and Methods

### Ethics statement

The study protocol and data collection were approved by the Institutional Ethics Committee for Clinical Research and Animal Trials of the First Affiliated Hospital of Sun Yat-sen University (Ethical Approval No: [2026]062). The requirement for informed consent was waived by the ethics committee because the dataset consists of de-identified surgical training videos and metadata, ensuring participant privacy and posing minimal risk.

### Anastomosis procedure

**Fig. 2:**
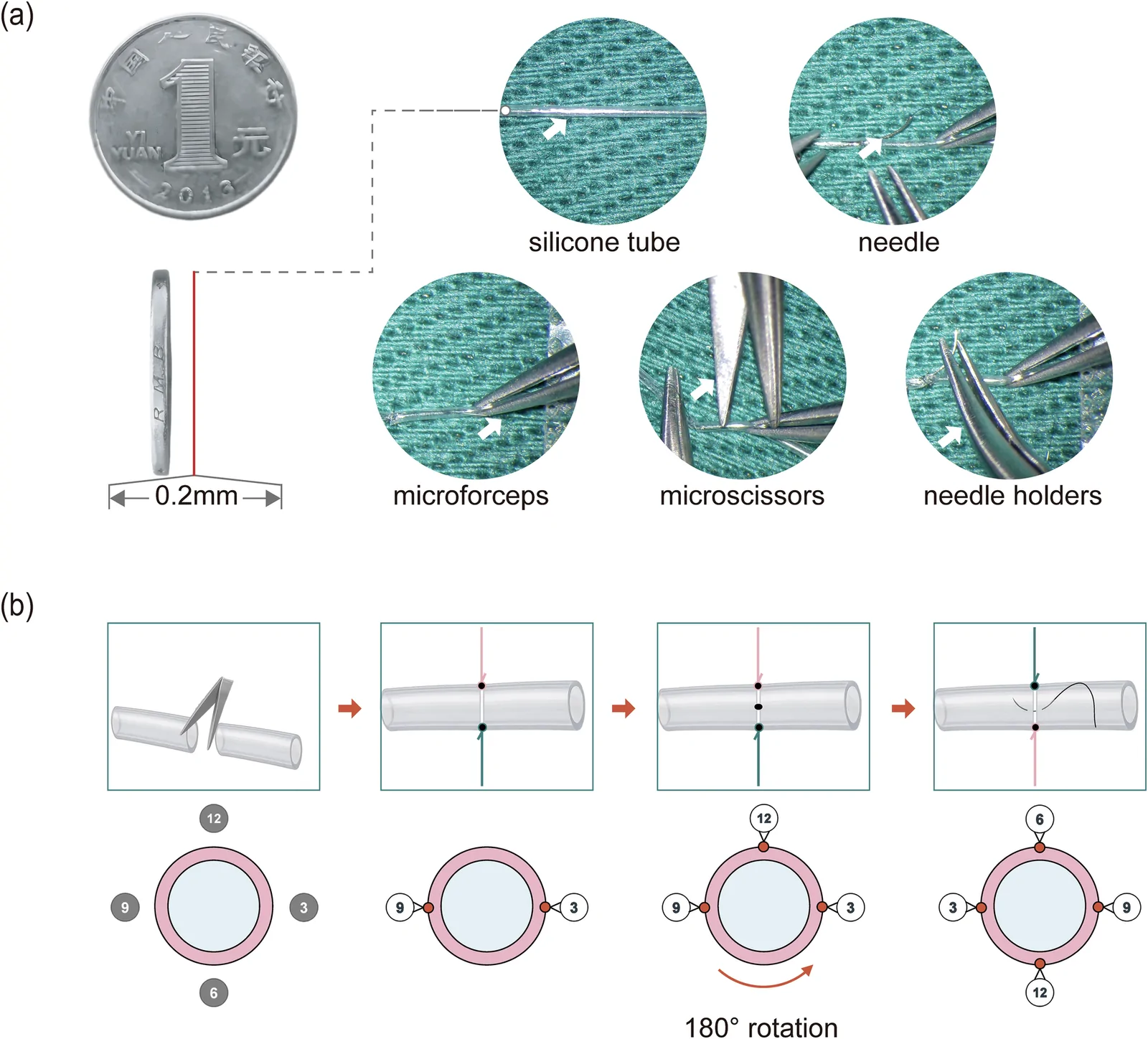
Supermicrosurgical instrumentation and anastomosis technique. (**a**) Essential supermicrosurgical instruments demonstrated on a 0.2 mm silicone tube model including microsurgical needles, microforceps, microscissors, and needle holders. Arrows indicate instruments. **(b)** The schematic illustrates the four-quadrant suturing method. Sutures are first placed at the 3 and 9 o’clock positions, followed by a third suture at the 12 o’clock position. After 180° rotation, the final suture is placed at the 6 o’clock position.

The surgical instruments used for anastomosis are shown in Fig. 2a. Core supermicrosurgical tools include microforceps, needle holders, a needle, and microscissors. Silicone tubes with a 0.2 mm diameter were employed to simulate blood vessels. The standardized anastomosis protocol proceeded as follows (Fig. 2b). After timing began, the operator transected the silicone vessel with microscissors to model a vessel injury. Then, the two tube ends were aligned to achieve precise end-to-end apposition. The first and second sutures were placed at the 3 o’clock and 9 o’clock positions and were then tightened. A third suture was placed at the 12-o’clock position. The anastomosis was next rotated 180° to expose the posterior wall, and a last suture was placed at the 6-o’clock position. Every suture was secured with three knots, and the suture tails were trimmed to an appropriate length. The bite distance from the vessel edge was generally twice the wall thickness, and the spacing between sutures was approximately twice the edge distance.

### Data collection

A total of 457 anastomoses were performed, of which 452 were successfully recorded; five were excluded because of file corruption caused by storage-device failure. Original recordings were obtained using the OMS3500 operating microscope (ZUMAX Medical Instruments Co., Ltd., Suzhou, Jiangsu Province, China). All videos were captured at 3,840 × 2,160 resolution and 30 frames per second. Silicone tubes with a diameter of 0.2 mm were used to simulate small blood vessels. To preserve the visual content, no image preprocessing, such as denoising, color adjustment, or compression beyond camera defaults, was applied; this ensured that all potentially important procedural details were retained for later image analysis and model training. Audio tracks were removed due to substantial ambient noise in the original recordings. During both device export and frame extraction, videos and images were automatically anonymized, ensuring that the final dataset contained no personally identifiable information and that individual participants could not be traced.

### Video processing and frame extraction

We constructed the Microsurgical Silicone Tube Anastomosis (MSTA) dataset from the collected operative videos. Non-operative segments immediately before and after each procedure were excised with LosslessCut (version 3.64; Mikael Finstad; https://github.com/mifi/lossless-cut). Trimming time points were jointly defined by an image-processing expert and a microsurgical specialist. From the trimmed clips, FFmpeg (version 7.1.2; FFmpeg Developers, https://ffmpeg.org/) was used to extract frames at 1 frame per second. All extracted frames were saved as samples within their corresponding folders.

### Annotation of frame data

**Fig. 3:**
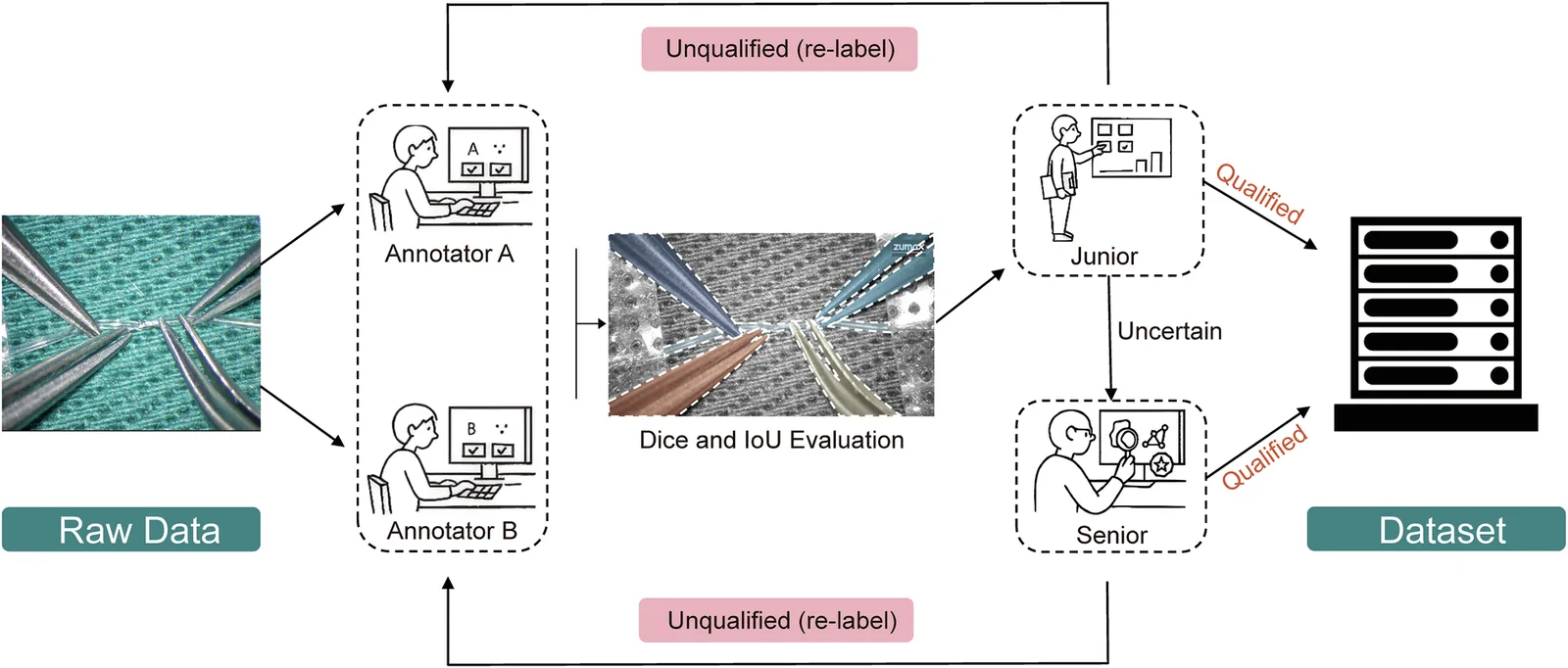
Data annotation workflow. Dual independent labeling process with quality control. Two annotators labeled the images, and then evaluated them using Dice coefficient, IoU and Cohen’s kappa metrics. The senior examiner adjudicated indeterminate cases.

Annotations were performed with LabelMe (version 5.8.3; Kentaro Wada; https://github.com/wkentaro/labelme). The annotation, review, and submission workflow is shown in Fig. 3. The annotation protocol defined five object classes: forceps, holder, scissors, needle, and tube. Objects were delineated using polygon contours along their visible boundaries. For instruments partially obscured by the translucent silicone tube, such as microsurgical needles during suturing, annotators were instructed to delineate the full instrument contour as accurately as possible. For small objects such as needles, annotators zoomed in during labelling to improve boundary precision. When only a small portion of an object appeared in a given frame, adjacent frames from the same video sequence were reviewed to confirm the object category. For motion-blurred objects, adjacent frames and the original video were similarly reviewed, and such objects were annotated when the category and approximate boundary could still be reliably determined in the target frame. For overlapping tools, each object was annotated separately according to its visible boundary and class label. Reflections and shadows were not annotated as object regions.

Prior to annotation, a reviewer provided standardized training to two junior annotators covering fundamental concepts of microsurgical anastomosis, key operative steps, instrument identification, and detailed labelling specifications. Inter-annotator agreement was evaluated using the Dice coefficient, Intersection over Union (IoU), and Cohen’s kappa. Agreement assessment was conducted in two rounds. In the first round (pre-annotation), 100 images were selected and independently annotated by both annotators using the same guidelines. Dice coefficient, IoU, and Cohen’s kappa were calculated from the paired annotations to quantify baseline consistency. The reviewer then audited this pilot set, corrected identified errors, and reconciled common sources of disagreement to further refine and standardize the annotation rules. Before formal annotation commenced, an additional 100 images were randomly sampled from the full dataset and reserved as an agreement audit set. During formal annotation, both annotators independently labeled the reserved audit set, whereas the remaining images were annotated by one of the two annotators. Two junior reviewers performed ongoing quality checks throughout the annotation process, and any uncertain or disputed cases were escalated to a senior reviewer for final adjudication. After completion of the full dataset, Dice coefficient, IoU, and Cohen’s kappa were recalculated on the reserved 100-image audit set to quantify final inter-annotator agreement following guideline refinement and process-level quality control.

## Data Records

**Fig. 4:**
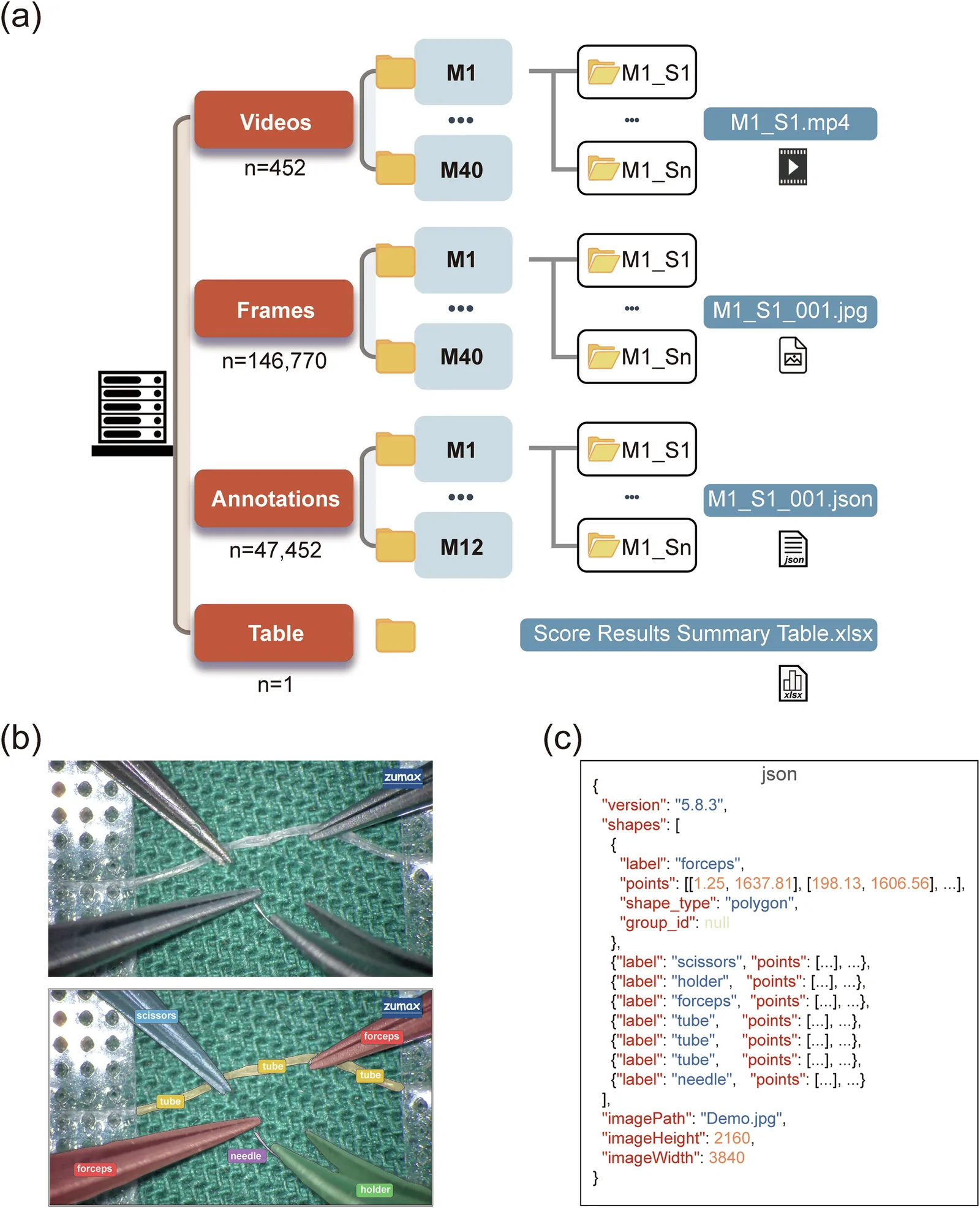
Dataset organization and annotation-file structure. **(a)** Organization of the deposited dataset, including Videos, Frames, Annotations, and Table. **(b)** Representative raw frame and corresponding LabelMe annotation overlay showing polygon-based labels for surgical instruments and tubes. **(c)** Representative excerpt of a LabelMe JSON annotation file, showing object labels, polygon coordinates, shape type, image filename, and image dimensions.

The dataset has been deposited in Science Data Bank (https://www.scidb.cn/) and is available under the https://doi.org/10.57760/sciencedb.31422^30^. The repository is organized into four components: Videos, Frames, Annotations, and Table (Fig. 4a). Together, these files provide the raw operative recordings, extracted image frames, segmentation annotations, and score information corresponding to each video. The Videos, Frames, and Annotations folders follow a consistent microscope and procedure-based naming scheme. Microscope identifiers are denoted as M1–M40. Procedures recorded under each microscope are numbered sequentially in the format Mx_Sy, where x represents the microscope identifier and y represents the procedure number for that microscope. For example, M1_S1 refers to the first anastomosis procedure recorded under microscope M1.

The Videos folder contains the original operative recordings in MP4 format. Each video corresponds to one complete simulated supermicrosurgical anastomosis procedure. Video files are named by procedure identifier, such as M1_S1.mp4. The Frames folder contains still images, extracted from the operative videos and saved in JPG format. In total, 146,770 images are included. Frame filenames follow the format Mx_Sy_FrameNumber. For example, M1_S1_001.jpg indicates the first extracted frame from procedure M1_S1. The Annotations folder contains segmentation annotations in LabelMe JSON format. A total of 47,452 images from the M1–M12 subset have been provided with corresponding annotation files. Each JSON file shares the same filename stem as its paired image; for example, M1_S1_001.json corresponds to M1_S1_001.jpg. The annotated categories include tube, forceps, holder, scissors, and needle. Each annotation file records polygon-based segmentation coordinates, object labels, image filename, image dimensions, and standard LabelMe metadata. Representative examples of the annotation overlay and LabelMe JSON file structure are shown in Fig. 4b and c. The Table directory contains the results in the file: Score Results Summary Table.xlsx. This table links each procedure identifier to the corresponding microscope number, procedure number, completion time, expert-derived score, and final total score. The main columns are Folder Name, M, S, Time Spent (seconds), Average Judges’ Score, and Total Score. Folder Name records the procedure identifier in the Mx_Sy format; M indicates the microscope identifier; S indicates the sequential procedure number; Time Spent (seconds) records the procedure duration; Average Judges’ Score records the mean expert score; and Total Score records the final score calculated according to the scoring rules described in the Methods section.

## Data Overview

### Overview of image and annotation counts

The MSTA dataset contains 452 complete silicone tube anastomosis procedures, comprising 40.77 h of original video and 146,770 extracted frames. Among these frames, 47,452 were manually annotated for segmentation, representing 32.33% of all images. All procedures were performed on 0.2 mm silicone tubes as vessel models. Using surgical microscopes at 20–50 × magnification and 11-0 nylon sutures, 571 participants from 158 hospitals completed simulated vascular anastomoses on silicone-tube vessel models.

**Fig. 5:**
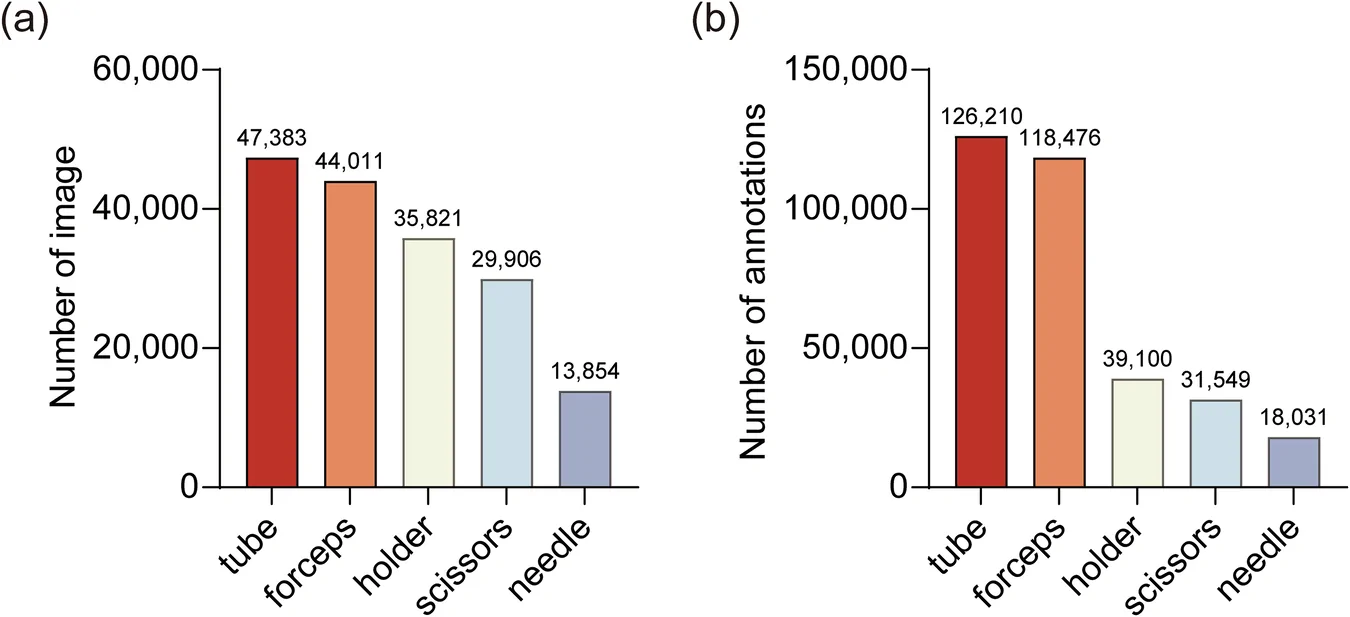
Distribution of annotated categories in the dataset. **(a**) Number of annotated frames containing at least one instance of each category. Counts are not mutually exclusive because one frame may contain multiple categories. **(b**) Number of annotations for each instrument category.

Table 1 summarizes the dataset composition and file sizes. The original video files occupy 178.78 GB, while the extracted frame images occupy 200.96 GB. The segmentation annotations are stored in LabelMe JSON format, with a total size of 0.55 GB. Figure 5a presents the frame-level distribution of annotated categories in the 47,452 annotated frames from the M1–M12 subset. Frame-level counts indicate the number of frames containing at least one annotation for a given category. Because multiple categories could appear in the same frame, these counts are not mutually exclusive. Tube and forceps were the most frequently annotated categories, appearing in 47,383 and 44,011 frames, respectively. Holder, scissors, and needle appeared in 35,821, 29,906, and 13,854 frames, respectively. Figure 5b shows the distribution of annotation instances. Across all LabelMe files, tube and forceps also accounted for the largest numbers of polygon annotations, with 126,210 and 118,476 instances, respectively. The remaining categories included 39,100 holder, 31,549 scissors, and 18,031 needle instances.

**Table 1:** Composition and statistics of the supermicrosurgical dataset.

Data Record	File format	Number of files	Size
Videos	MP4	452	178.78 GB
Frames	JPG	146,770	200.96 GB
Segment Annotations	JSON	47,452	0.55 GB
Table	XLSX	1	29.30 KB

### Participant demographics

**Fig. 6:**
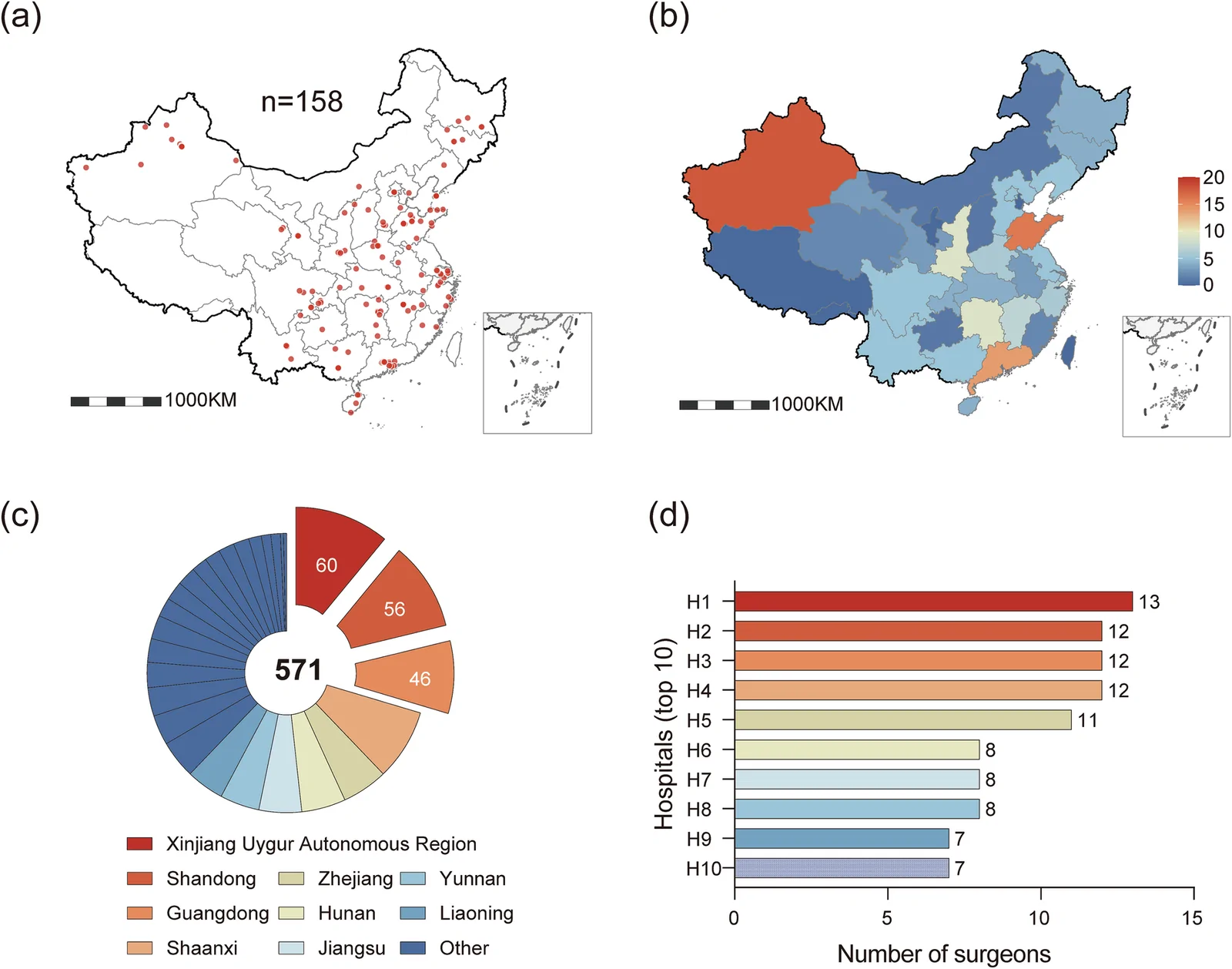
Geographical distribution of participating hospitals and operators. **(a)** Geographical location of 158 participating hospitals across the country, and the red dots represent the location of hospitals in 28 provinces and autonomous regions. **(b)** Regional distribution of hospital participation intensity, the color gradient (blue to red, scale 0-20) represents the number of participating hospitals in each province, and darker red indicates a greater number of participating hospitals. **(c)** Provincial distribution of 571 matching operators, the pie chart shows the proportion of operators in different provinces, and the “other” category (blue part) represents the combined share of provinces each contributing a relatively small number of operators. **(d)** Top 10 hospitals ranked by number of participating operators.

Operators in the MSTA dataset were recruited from 158 hospitals across 28 provinces in China (Fig. 6a). Participating hospitals were distributed across most regions of the country. The Xinjiang Uygur Autonomous Region had the highest number of hospitals (18, 11.3%), followed by Shandong (17, 10.7%) and Guangdong (14, 8.8%). Hunan and Shaanxi each had 9 participating hospitals (5.7%) (Fig. 6b). Public hospitals accounted for 87% of sites and private hospitals for 13%. Overall, hospitals in eastern China (e.g., Shandong, Zhejiang, Jiangsu) and southern China (e.g., Guangdong, Hunan) were more concentrated and showed higher participation rates. Across all participating hospitals, 571 operators performed anastomoses. Provinces with the largest numbers of operators were the Xinjiang Uygur Autonomous Region (60, 10.5%), Shandong (56, 9.8%), Guangdong (46, 8.1%), Shaanxi (45, 7.9%), and Zhejiang (29, 5.1%) (Fig. 6c). A complete list of operators by province is provided in Table 2.

**Table 2:** Provincial distribution of participants in the supermicrosurgical anastomosis dataset.

Province	n	%	Province	n	%
XUAR	60	10.51	Shanghai	15	2.63
Shandong	56	9.81	Hainan	15	2.63
Guangdong	46	8.06	Hebei	12	2.10
Shaanxi	45	7.88	Jiangxi	12	2.10
Zhejiang	29	5.08	Beijing	12	2.10
Hunan	28	4.90	GXZAR	10	1.75
Jiangsu	27	4.73	Jilin	10	1.75
Yunnan	25	4.38	IMAR	10	1.75
Liaoning	24	4.20	Heilongjiang	10	1.75
Sichuan	24	4.20	Fujian	8	1.40
Henan	24	4.20	Anhui	6	1.05
Gansu	19	3.33	Qinghai	6	1.05
Hubei	18	3.15	Shanxi	2	0.35
Chongqing	16	2.80	Guizhou	2	0.35

Abbreviations: n, number of participants; XUAR, Xinjiang Uygur Autonomous Region; GXZAR, Guangxi Zhuang Autonomous Region; IMAR, Inner Mongolia Autonomous Region.

Operator participation varied across hospitals. The median number of operators per hospital was 2 (IQR 2–6), with a pronounced long-tailed distribution. Five hospitals had 10 or more operators, and 52 hospitals had at least 5. Eighty-four hospitals had one or two operators, including 18 hospitals with only one operator. The top 10 hospitals collectively contributed 98 operators (16.9% of the total), and the top 20 accounted for 162 operators (27.9%) (Fig. 6d). In general, a small number of core centers hosted many operators, while most hospitals had one or two, consistent with a core–periphery pattern.

### Performance assessment

We developed a new scoring system based on the MARS10^31^ microscopic anastomosis scoring protocol, incorporating the time factor score and tissue processing conditions that were not included in the original protocol. The maximum possible score was 105, covering four domains: accuracy of suture placement, approximation of vessel stumps, stability of needle holder and needle manipulation, and efficiency of operative time. Table 3 presents the full scoring criteria. The Average Judges’ Score was calculated as the mean technical score assigned by the three microsurgical experts. The Total Score was then calculated as the Average Judges’ Score plus the time-efficiency score, with a maximum possible score of 105. Procedures not completed within 600 seconds were disqualified and assigned a Total Score of 0. Higher scores indicate superior microsurgical anastomosis skill.

**Table 3:** Scoring rubric for microsurgical anastomosis assessment.

Domain	Criterion	Scoring Details	Points
Time Efficiency	Completion time	Score = 20 − (t/60); t = time in seconds	20
Technical Compliance	Procedural standards	a) Number of sutures meets protocol requirements b) Suture tail length appropriate (not excessive)	25
Technical Proficiency	Manual dexterity	a) Gentle tissue handling b) Accurate needle placement c) Stable hand movements d) The operation is smooth	30
Anastomosis Quality	Structural integrity	a) Vessel walls properly aligned without gaps b) Uniform spacing between sutures c) Consistent distance from vessel edge	30
Total Score			105

Procedures not completed within 600 seconds were disqualified and assigned a Total Score of 0.

**Fig. 7:**
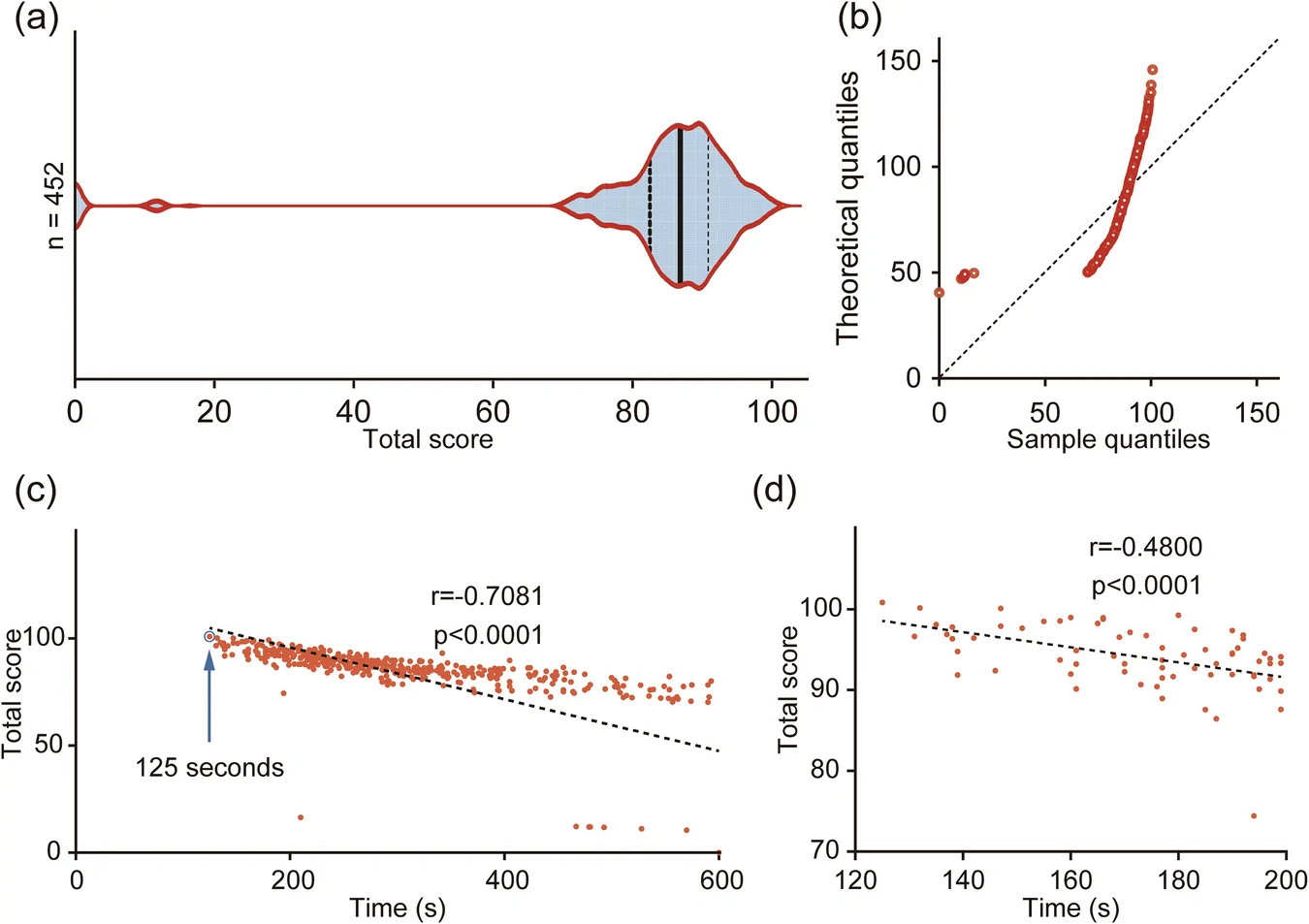
Performance scores and completion time analysis of microsurgical anastomosis procedures. **(a)** Violin diagram shows the distribution of procedure-level final Total Scores. **(b)** A Q-Q plot was used to compare the sample data with a theoretical distribution and assess goodness-of-fit. **(c)** Scatter chart shows the correlation between the completion time and the total score of all procedures. **(d)** Correlation analysis of the operation subsets with a completion time of less than 200 seconds.

Figure 7a shows the distribution of procedure-level final Total Scores. The median score was 86.83 (interquartile range: 82.51–90.83), the mean ± standard deviation was 81.95 ± 20.79, and the range was 0–100.83 (out of a possible 105). Using threshold criteria, 135 of 452 procedures (29.87%) scored ≥ 90, whereas 20 of 452 (4.42%) received zero due to time expiration resulting in disqualification. A normal Q-Q plot was used to visually assess the distribution of procedure-level final total scores against a theoretical normal distribution (Fig. 7b). The points deviated from the reference line, indicating that the score distribution was not normally distributed. This deviation was consistent with the scoring structure of the dataset, including a small group of zero scores caused by time expiration and clustering of observations toward the high-score range. Therefore, the score distribution was interpreted descriptively rather than assumed to follow a normal distribution. Overall, the score distribution indicates that most participants achieved intermediate to advanced microsurgical proficiency.

### Anastomosis time

The total time from the start to the completion of the anastomosis was defined as the anastomosis time. The start time was recorded when the operator received the instruction to begin, and the end time was recorded when all suture tails were cut and the participant informed the timekeeper that the procedure was finished. During the procedure, operators were required to place four sutures, with three knots for each suture. The silicone tube could not be touched before the official start instruction. The operation was terminated if the procedure was not completed within 10 minutes. The median anastomosis time was 280 seconds (range: 125–600 seconds) (Fig. 7c). Completion time showed a strong negative correlation with the total score (Pearson r = −0.708, 95% CI: −0.751 to −0.659; p < 0.001), indicating that shorter times were associated with higher performance. Among procedures completed within 200 seconds (n = 68), this negative association remained statistically significant but was weaker than in the full dataset: the Pearson correlation coefficient decreased from −0.708 to −0.480 (r²: 0.50 to 0.23), and Spearman’s ρ decreased from −0.880 to −0.498 (both p < 0.001). Similarly, the linear slope changed from −7.24 points per minute (95% CI: −7.91 to −6.57) to −5.61 points per minute (95% CI: −8.14 to −3.09) (Fig. 7d). These results indicate that within this time range, longer completion time remained associated with lower total scores, although the effect was reduced.

### Distribution of operators by institutional background

Hospital type was retained as descriptive metadata to characterize the composition of the dataset. The dataset included operators from both public and private hospitals, reflecting heterogeneity in institutional backgrounds among participants. No comparative conclusions regarding performance by hospital type were drawn, because the study was designed as a dataset descriptor rather than as an institutional performance comparison.

## Technical Validation

### Annotator consistency

There was high inter-annotator agreement across both rounds. For the pre-annotation pilot set (n = 100), the corresponding values were 0.9777 (Dice coefficient), 0.9571 (IoU), and 0.9361 (Cohen’s kappa). For the reserved audit set assessed after dataset completion (n = 100), the corresponding values were 0.9821 (Dice coefficient), 0.9668 (IoU) and 0.9456 (Cohen’s kappa).

### Deep learning model training

We trained and compared deep learning models to evaluate the suitability of the annotated dataset for learning and generalization. Multi-model benchmarking facilitates comprehensive assessment of algorithm performance under complex microscopic conditions and provides a baseline for future model improvement.

For technical validation, we evaluated two complementary segmentation frameworks, YOLOv12^32^ and nnU-Net. YOLOv12 was selected as a representative real-time segmentation model from the YOLO family, suitable for assessing whether the annotated frames could support efficient detection and segmentation of small surgical instruments. nnU-Net^33^ was selected as a self-configuring medical image segmentation framework, providing a second validation approach with a different model design and training pipeline. Evaluating both models allowed us to assess the usability of the annotation set across different segmentation frameworks.

For technical validation, the annotated images were split at the procedure level rather than the individual-frame level to prevent information leakage from temporally adjacent frames within the same video. Procedures recorded under microscopes M1–M9 were assigned to the training set, those under M10 and M11 to the validation set, and those under M12 were held out as the test set. The same data split and ground-truth annotations were used for both YOLOv12 and nnU-Net, and all Dice coefficient and IoU values reported in this study were computed on the held-out test set (M12). For YOLO, we used the YOLOv12n-seg variant initialized from pretrained weights (yolo12n-seg.pt) rather than training from scratch using a YAML configuration. For nnU-Net, we used version 2 with the 2D configuration (nnUNetTrainer__nnUNetPlans__2d, fold 0), trained from scratch using its self-configuring pipeline. Consistent results across these two architectures support the technical usability of the annotation set for downstream segmentation model development, although further evaluation on independent external datasets will be needed to assess broader generalizability.

**Fig. 8:**
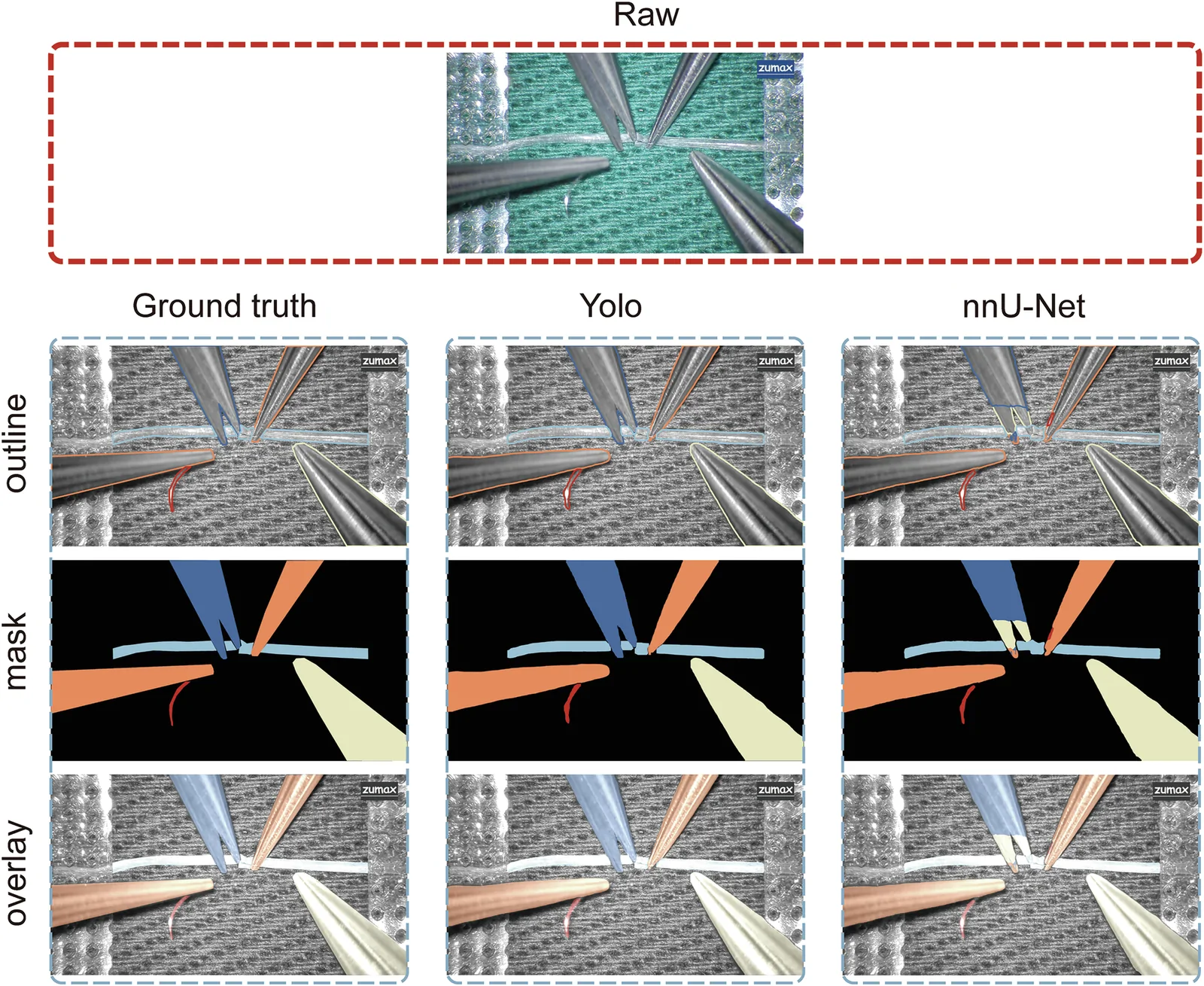
Comparison of the detection and segmentation performance of different models. Top figure: raw surgical frames. Bottom rows: ground truth, YOLO predictions, and nnU-Net predictions. Each set shows outline boxes (top), segmentation masks (middle), and overlays (bottom). Different colors represent different instrument classes.

Class-wise analysis showed that segmentation performance varied across object categories. Both models achieved high Dice and IoU values for larger or more frequently visible objects, such as scissors, forceps, holder, and tube. In contrast, needle segmentation showed lower performance, likely because needles are small, thin, partially occluded, and less frequently visible in the held-out test images. Table 4 shows the full results. Visual comparisons of different models are shown in Fig. 8.

**Table 4:** Performance evaluation of nnU-Net and YOLO models in instrument segmentation.

Model	Metric	Forceps	Holder	Needle	Scissors	Tube
YOLOv12	Dice coefficient	0.8351	0.9266	0.4178	0.9238	0.8350
	IoU	0.7343	0.8867	0.3118	0.9115	0.7250
nnU-Net	Dice coefficient	0.9743	0.9549	0.4687	0.9912	0.9434
	IoU	0.9506	0.9353	0.3880	0.9825	0.8940

IoU, Intersection over Union.

### Challenging scenarios in surgical instrument recognition

**Fig. 9:**
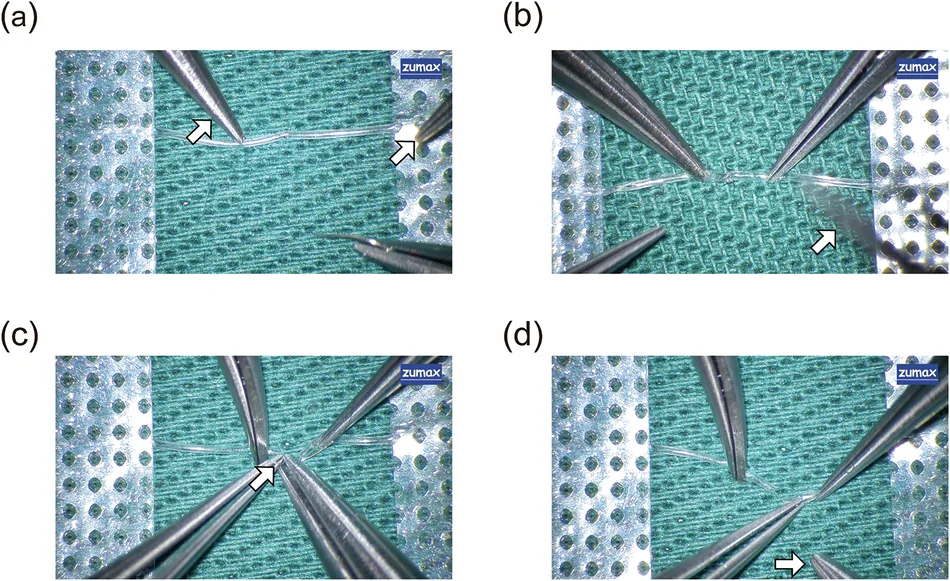
Typical challenging scenarios encountered in the identification of microsurgical instruments. **(a)** High morphological similarity between the tips of forceps and scissors (arrow), complicating distinction. **(b)** The motion blur artifact (arrow) reduces the clarity of the instrument features. **(c)** The tiny suture needle (arrow) is partially covered by the surrounding instruments. **(d)** High magnification (arrow) causes the field of view to be limited to the distal tip of the instrument.

There are a number of inherent difficulties in accurately identifying the instruments used in microsurgical anastomosis (Fig. 9). First, as shown in Fig. 9a, instrument tips show remarkable morphological similarity under high magnification. The tip geometries of scissors and forceps are almost identical, which presents significant challenges for automated recognition systems. Second, fast-moving instruments produce motion blur artifacts that impair visual clarity. Figure 9b is an example of this phenomenon, where moving instruments exhibit blurry contours and lack discriminative features, greatly impairing detection performance. Third, a combination of small object size and partial occlusion leads to significant recognition challenges. As Fig. 9c illustrates, the suture needle is difficult for detection algorithms to recognize due to its small size and partial concealment by nearby instruments and the silicone tube. Fourth, high magnification results in insufficient instrument visualization; as Fig. 9d illustrates, only the instrument tips are visible, lacking the contextual cues and morphological features required for reliable classification.

### Advantages and limitations of the dataset

Publicly available microsurgical datasets remain scarce. Existing representative resources, such as MISAW^28^ and CUHK-MRG^29^, provide surgical videos and, in some cases, robotic kinematic data for microvascular anastomosis procedures. A descriptive comparison of these datasets is presented in Table 5. To our knowledge, the MSTA dataset constitutes the largest publicly available collection of simulated microsurgical anastomosis procedures and the first specifically devoted to 0.2-mm silicone-tube anastomosis for supermicrosurgery. Its principal strength lies in the integration of complete 4 K operative videos, extracted high-resolution frames, polygon-based segmentation annotations, procedure-level metadata, and expert-derived performance scores. These resources may support the development and evaluation of computer vision models for surgical instrument segmentation, object detection, workflow analysis, and phase recognition. The dataset may also serve as a reference resource for future investigations of robotic supermicrosurgery and human performance in technically demanding microsurgical tasks. Robotic platforms such as Symani and MUSA reflect the growing interest in systems capable of enhancing stability and precision during microsurgical procedures, and datasets of this nature may facilitate the development of perception modules for future assistive systems.

**Table 5:** Comparative characteristics of publicly available microsurgical anastomosis datasets.

Characteristic	CUHK-MRG	MISAW	MISRA (based on MISAW)	MSTA (present study)
**Acquisition year**	2021	2021	2024	2025
**Number of cases**	24	27	27	452
**Vessel diameter (mm)**	NR	NR	NR	0.2
**Number of operators**	2	6	6	571
**Number of centers**	2	1	1	158
**Performance assessment**	No	No	No	Yes
**Total video duration (h)**	NR	NR	NR	40.77
**Native resolution (pixels)**	NR	960 × 540	960 × 540	3,840 × 2,160
**Annotation granularity**	Frame-wise gestures with kinematics	Multi-level workflow (phase/step/activity) with kinematics	Pixel-level segmentation	Pixel-level segmentation
**Pixel-level segmentation**	No	No	Yes	Yes
**Extracted frames (n)**	0	0	2,999	146,770
**Annotated images (n)**	0	0	2,999	47,452
**Number of label classes**	-	-	6	5
**Thin-object annotation**	No	No	Yes (needle, wire)	Yes (needle)
**Multi-modal data**	Video + kinematics	Video + kinematics	RGB + masks	Video + RGB + masks
**Surgical platform**	Robot-assisted	Robot-assisted	Robot-assisted	Manual microsurgery
**Clinical data inclusion**	No	No	No	No

NR, not reported; Bold text indicates values from the present study.

Several limitations should be acknowledged. First, the procedures were performed on simulated 0.2 mm silicone tubes rather than living vessels and therefore did not capture the full range of tissue properties, bleeding, and physiological variability encountered in clinical surgery. Second, although the dataset encompasses a large volume of raw video and frame data, segmentation annotations were generated for only a subset of frames, as high-quality polygon annotation was labor-intensive. Third, data collection was conducted exclusively in China and did not include operators or hospitals from other regions, which may limit the generalizability of models trained solely on this resource. Fourth, the present release focuses primarily on raw visual data, segmentation annotations, metadata, and expert-derived scores; additional derived labels, such as detailed surgical phase annotations or motion-based performance metrics, could further enhance its utility in future work. Finally, the performance scores warrant particular caution in interpretation. Although the scoring rubric was derived from MARS10, it was adapted for the present simulated 0.2 mm supermicrosurgical anastomosis task and has not been independently validated as a formal skill-assessment instrument. Moreover, the original MARS10 scale has been reported to exhibit a ceiling effect among high-performing individuals. This concern is potentially amplified in the present dataset, given that participants were self-selected and many were experienced surgeons. The 10-minute time limit may have further reduced the representation of prolonged or technically challenging procedures. Collectively, these factors may introduce selection bias and score compression in the upper range. Accordingly, the performance scores are best regarded as expert-derived descriptive metadata and reference labels rather than definitive measures of surgical proficiency. Downstream studies employing these scores for skill assessment or model training should account for these limitations.

## Usage Notes

This study establishes the first 0.2 mm silicone tube anastomosis dataset, with the aim of accelerating the development and application of AI in microsurgery. Human performance in microsurgery is limited by physiological hand tremor, physiological fatigue, and constraints in perceptual accuracy^34^, making it difficult to sustain stable submillimeter precision during prolonged procedures. In addition, manually controlled robotic systems for single anastomosis are associated with significantly longer operative times than conventional surgeon-performed techniques^35^. The MSTA dataset is intended to enable AI models to learn from high-level human performance, acquire key capabilities such as suture path planning, tissue recognition, and force control, and to provide a foundation for autonomous anastomosis in robotic microsurgery.

In addition, the MSTA dataset has important implications for supermicrosurgical education and skills assessment. Each high-definition video records the complete anastomosis process. With frame annotation, the individual suturing steps can be systematically segmented, allowing learners to clearly observe key technical details and providing a standardized training resource for microsurgical curricula. Notably, the dataset includes a high-quality 0.2 mm anastomosis case in which the operator completed end-to-end anastomosis within 125 seconds, demonstrating both speed and precision at the upper limit of current human performance. Such exemplary cases may provide a foundation for future studies toward autonomous robotic microsurgery.

In future work, we will continue to expand the dataset by adding manually annotated images and additional anastomosis models, including rat tail vascular anastomosis and clinical anastomosis data. Subsequent efforts will incorporate multimodal synchronized data, such as video, motion, and audio signals. These developments will further strengthen the MSTA dataset as a core resource for research and innovation in supermicrosurgery.

## Data Availability

The MSTA dataset described in this Data Descriptor is publicly available from Science Data Bank under the Creative Commons Attribution 4.0 International license (CC BY 4.0) at https://doi.org/10.57760/sciencedb.31422^30^.

## Code availability

The MSTA dataset can be used without additional custom code. All tools employed in the experiments are publicly available under open-source licenses. The segmentation masks can be generated directly using LabelMe (https://github.com/wkentaro/labelme) and its built-in mask conversion utilities. The model implementations used for technical validation are available at: nnU-Net: https://github.com/MIC-DKFZ/nnUNet YOLOv12: https://docs.ultralytics.com/models/yolo12.
